# Plans and prospects for the 2020s: Beyond peak health?

**DOI:** 10.1371/journal.pmed.1003075

**Published:** 2020-02-25

**Authors:** The PLOS Medicine Editors

**Affiliations:** Public Library of Science, San Francisco, California, United States of America and Cambridge, United Kingdom

## Abstract

The PLOS Medicine editors discuss prospects for health and development in the coming decade.

What will the new decade bring? The 2020s are still new and unfamiliar, and difficult unresolved issues continue from the recent past. But if plans come to fruition this should be a transformational period for the health and wellbeing of the world’s population. The United Nations’ 17 Sustainable Development Goals (SDGs) are carefully drawn up and ambitious (encompassing “no poverty”, “zero hunger”, and other important aspirations among the plethora of goals and targets), and the organization proclaims that a “decade of ambitious action” is needed to “deliver the goals by 2030” [1]. Nor is there any lack of attention to individual disease areas among agencies, with WHO’s End TB strategy projected, with periodic updates, to run until 2035 [2], for instance, and plans formulated to leverage new and existing therapeutic options to eliminate hepatitis B and C as public health threats in a similar timeframe [3].

Behind the headlines, executive summaries and good intentions, it is apparent that such plans, bold and eyecatching as they are, are likely to fall short. This should not be viewed as a shortcoming of supranational bodies like WHO, which are to be commended for leading health agendas in bringing together donors, researchers, health workers and others to deploy coordinated efforts against the major global health challenges [4]. Although WHO has not lacked critics, an agency with renewed vigour and purpose will be much needed to identify and orchestrate decisive action against new threats—with a new coronavirus outbreak emerging in Asia at the time of writing—as well as familiar and intractable ones (e.g., the long-imagined eradication of polioviruses).

The global population itself is one of the major challenges to ambitions for improved health and progressive development. With the population forecast to grow from the current estimated 7.7 billion to 8.5 billion by 2030 [5], it would be foolish not to expect ongoing pressures on health systems, resources and the environment. Substantial population growth is anticipated in parts of Asia, sub-Saharan Africa and the Americas, and in these areas especially governments and agencies will need to work effectively together and redouble efforts to avoid the existence, in 2030, of populations and regions forgotten by the SDGs. As the populations of the richest countries age, and probably diminish in size, large-scale migration can be expected as a continuing theme, and one calling for ongoing attention in view of uncertainties for the health of refugees and migrants in many jurisdictions (the topic of a *PLOS Medicine* special issue to be published this March).

The trajectory and effects of climate change will perhaps be the defining issue of the coming decade. Most climate scientists and some leaders have for several years been strident in the view that decisive and immediate action is needed to bring about wholesale reductions in fossil fuel emissions and use of other pollutants, along with mitigation of their effects on people and the environment. Yet there is an apparently disastrous lack of unanimity on where action needs to take place, by whom and even, remarkably, when. How can the doctrine of economic growth, on which many of the tangible and psychological elements of societies depend, be reconciled with the accompanying destructive environmental hazards? The times of global consensus and compromise embodied in the Paris Climate agreement of 2016, seeking to limit the increases in global temperatures and hence their adverse effects [6], have given way to a period of uncertainty and disunity—an era of “me” rather than “we”.

Recall “peak oil”, the notion that oil production—yielding fuels essential for most forms of transportation to this day, and with many other apparently indispensable industrial uses—would reach a maximum and thereafter decline owing to diminishing success in exploration [7]. Although it seems that a global peak has not yet, and may never be, reached, one can imagine that the consequences of progressive oil scarcity could be dramatic, leading to challenging readjustments of societies and economies to develop alternative sources of energy and reduce reliance on environmentally damaging fuels. Today, it seems ludicrous that warnings of a possible peak of oil production in the late 20^th^ century did not stimulate research and the development of large-scale alternatives alongside the quest for more oilfields.

Despite the positive vision embodied by the SDGs, could “peak health” have already been reached? Although the analogy with peak oil may be debatable, there are signs that life expectancy in the United States and United Kingdom has reached a plateau, and may be declining [8]. Health inequalities abound. It is anticipated that improved disease prevention and health provision in developing countries will continue to deliver improvements in life expectancy and reductions in life-years lost to disability and ill health but, in all countries, new health challenges will undoubtedly emerge. We must hope that progress in population health does not slip into reverse gear in the coming decades, driven by factors that could include the transition to non-communicable diseases, vaccine hesitancy, environmental stressors, and anticipated but unpredictable hazards such as antimicrobial resistance. When today’s leaders return to the paradigm of consensus from that of confrontation, high on their to-do lists should be to work together to find an area of convergence between economic progress, environmental stabilization and continued improvement in human health and wellbeing, all of which can be assessed with meaningful indicators. Must we wait until the gains in health made over recent decades are lost?
